# Comprehensive Pain Management in Total Joint Arthroplasty: A Review of Contemporary Approaches

**DOI:** 10.3390/jcm13226819

**Published:** 2024-11-13

**Authors:** Daniel N. de Souza, Nathan A. Lorentz, Lefko Charalambous, Matthew Galetta, Christopher Petrilli, Joshua C. Rozell

**Affiliations:** Department of Orthopedic Surgery, NYU Langone Health, New York, NY 10016, USA; daniel.desouza@nyulangone.org (D.N.d.S.); nathan.lorentz@nyulangone.org (N.A.L.); lefko.charalambous@nyulangone.org (L.C.); matthew.galetta@nyulangone.org (M.G.); christopher.petrilli@nyulangone.org (C.P.)

**Keywords:** total hip arthroplasty, total knee arthroplasty, multimodal pain management, standardization, analgesia

## Abstract

**Background:** Total hip and knee arthroplasties are among the most effective and widely performed procedures in modern medicine, providing substantial benefits to patients with end-stage osteoarthritis. These surgeries have transformed the treatment of degenerative joint disease, significantly enhancing functionality and quality of life for patients. Despite considerable advancements in surgical techniques and postoperative care, managing postoperative pain remains a major challenge, impacting both clinical recovery and patient satisfaction. The persistence of postoperative pain as a barrier to recovery underscores the need for improved pain management strategies. **Methods:** A comprehensive narrative review of the literature was conducted, focusing on the physiological mechanisms underlying surgical pain, the role of anesthesia techniques, and the development of multimodal pain management approaches used in total joint arthroplasty. This review emphasizes the components of modern multimodal strategies, which combine multiple pharmacologic and non-pharmacologic methods to address the various mechanisms of postoperative pain. **Results:** Current pain management strategies employ a dynamic, multimodal approach that covers the perioperative period. These strategies aim to optimize pain control while minimizing side effects. They incorporate a range of methods, including nerve blocks, non-opioid analgesics, opioids, and non-pharmacologic techniques such as physical therapy. However, evidence regarding the efficacy and optimal combinations of these interventions varies widely across studies. **Conclusions:** This variation has led to inconsistent pain management practices across institutions. To standardize and improve care, this paper presents the authors’ institutional pain management model, offering a potential framework for broader application and adaptation in the field of joint arthroplasty.

## 1. Introduction

Total hip and knee arthroplasty (THA, TKA) can dramatically improve the mobility, functionality, and quality of life for patients with severe arthritis. Over one million total joint arthroplasties (TJA) are performed annually in the U.S. and that number is expected to continue growing over the coming decade [1,2]. Over the last several decades, there have been significant innovations, including advancements in materials, implant designs, and cost-effectiveness [3].

In addition to improved patient outcomes, these innovations have contributed to reduced hospital lengths of stay, an increasingly important metric due to rising healthcare costs and evolving value-based reimbursement models. In the last decade, the average hospital stay for THA has decreased by over 50%, from three days in 2012 to 1.4 days in 2021. A similar trend has been observed for both unicompartmental and TKA during the same period, decreasing from 2.9 days in 2012 to 1.3 days in 2021 [4]. This trend is also not unique to the U.S., with similar observations reported based on data from the English National Health Service and in Asia [5,6].

Despite these improvements, the length of inpatient stay following TJA remains a significant contributor to national healthcare costs [5]. One analysis of nearly five million cases found that 39.6% of all TJAs in the U.S. resulted in hospital stays of three days or longer, and 10% of procedures led to stays exceeding four days [7]. These findings suggest that a considerable portion of TJA patients remain inpatient at least 25% longer than average, with a small minority staying more than 66% longer. Moreover, the same study showed that hospital costs increased by 5–8% for each additional night as an inpatient following TJA [7,8]. In 2016, total hospital costs for patients who remained inpatient for three days or more (n = 505,500) accounted for a 4.16 billion USD increase compared to a scenario where those patients had been discharged after two midnights in the hospital [7].

Predictive indicators for increased length of hospital stay following TJA have been extensively investigated, and it is well-understood that significant postoperative pain contributes to delayed discharge [9]. Previous studies have attempted to predict which patients will develop recovery-inhibiting pain, yet the predictive factors reported by these models are broad, unspecific, and yet to be validated [8,10,11,12,13]. In addition to the patient’s subjective perception of pain severity directly influencing discharge readiness, postoperative pain can deter patients from early ambulation, regaining range of motion, and mobilizing with physical therapy. Consequently, this can prevent timely discharge home. Pain-induced delays to beginning physical therapy can also increase a patient’s risk profile for developing venous thrombosis and other complications [14].

Pain has dramatic negative impacts on patient satisfaction, long-term outcomes, and likelihood of readmission [15], so managing patient expectations of pain associated with TJA remains critically important. The objective of this review is to summarize and evaluate contemporary approaches to pain control in TJA patients. We will first examine the physiological mechanisms of surgical pain and then outline a comprehensive strategy, based on the NYU Langone Health protocol, to mitigate pain and accelerate recovery.

## 2. Postoperative Pain: Surgical Causes and Mechanisms of Perception

### 2.1. Inflammatory Pain Response and Signaling Pathway

Soft tissue trauma during TJA leads to a local and systemic inflammatory cascade [16]. Insult to the local tissues causes the release of mediating compounds such as prostaglandins, cytokines, and other inflammatory markers that signal the presence of trauma to the immune system and initiate healing. These markers are subsequently detected by nociceptors which trigger afferent electrical signaling in peripheral nerves [17].

These afferent signals travel to the dorsal root ganglion (DRG) and the dorsal horn of the spinal cord where they synapse with secondary afferent interneurons and are modulated by a complex array of excitatory and inhibitory mechanisms. Secondary afferent neurons then project the processed somatosensory information to the brainstem of the supraspinal central nervous system (CNS) where it is further modulated and projected to other areas of the brain [18]. The signals are transmitted to the brainstem reticular formation, midbrain, thalamus, and cerebral cortex via two primary ascending pathways, the contralateral spinothalamic and spinoreticular pathways, where after higher central processing they are experienced as pain [19].

### 2.2. Hypersensitization and Neural Plasticity

The peripheral nociceptive neurons that sense the original inflammatory insult are highly plastic and become sensitized following the initial flood of inflammatory compounds. As a result, lower-intensity sensory inputs will subsequently trigger exacerbated pain responses [20]. The CNS demonstrates a similar type of plasticity in response to increased signaling from peripheral neurons, enhancing further signaling with ongoing nociceptive input. One mechanism driving this phenomenon involves the disinhibition of interactions between the primary CNS neurotransmitter glutamate and post-synaptic receptors, including N-methyl-D-aspartate (NMDA) receptors [21]. This type of sensitization is an adaptive mechanism to promote wound healing; however, it becomes maladaptive with severe or chronic post-surgical pain.

### 2.3. Neuropathic Pain

Postoperative pain may also result from surgically induced neuropathic pain due to direct trauma to peripheral nerves. Studies demonstrate that 10–40% of patients experience chronic neuropathic pain as a result of unintended neuronal trauma during surgery [22]. Clinically, neuropathic pain is characterized by persistent or radiating pains in the affected area which can be triggered or intensified by external stimuli, noxious and otherwise.

Peripheral nerve injury has also been associated with aberrant impulses from injured nerves and nearby uninjured nerves. This occurs because peripheral neurons that detect damage selectively amplify the gene expression for various voltage-gated cation channel subtypes following trauma [23]. Among the upregulated channel types are voltage-gated sodium channels (VGSC) and voltage-gated calcium channels (VGCC), which increase expression in the peripheral nerves and the DRG [24,25,26,27]. Heightened expression of cation channels leads to increased excitability, lowered activation thresholds, and amplified nociceptive signal transmission from peripheral nerves.

## 3. Systemic Pharmaceutical Options for Pain Control and Recovery

### 3.1. Opioids

Opioids function by binding to mu-opioid receptors in the CNS, competitively inhibiting pain signaling. As of 2019, opioids were prescribed to about 40% of U.S. knee osteoarthritis patients, and as of 2017, approximately one-third of TKA patients had taken opioids in the three months prior to their surgery [14,28,29]. Postoperative opioid prescriptions are standard for the vast majority of TJA pain control protocols because of their efficacy for reducing surgical pain [30].

However, minimizing postoperative opioid requirements for TJA patients is clinically advantageous due to harmful side effects associated with their chronic use. Common side effects of opioid consumption include tolerance, addiction, respiratory depression, and hyperalgesia, among others, and their over-prescription has resulted in one of the most severe public health crises in U.S. history [31,32].

Chronic opioid use prior to TJA has been associated with worse outcomes in terms of pain control thresholds and recovery [30]. Specifically, patients with pre-existing history of opioid use exhibit lowered pain tolerance, higher subjective pain scores, increased opioid consumption, reduced functionality, longer recovery times, lower overall satisfaction, and increased care costs when compared to opioid-naive patients [33,34,35,36]. Further, opioid-tolerant patients may also require higher dosages of postoperative opioid analgesia for sufficient pain control. As a result, one of the primary goals of multimodal pain control is to minimize the need for postoperative opioids, and why non-opioid therapy, when sufficiently effective, is preferred [37].

### 3.2. NSAIDs, Selective Cyclooxygenase Inhibitors, and Acetaminophen

NSAIDs such as naproxen, ibuprofen, and others work to reduce pain and inflammation by inhibiting cyclooxygenase 1 and 2 (COX-1/2), isoenzymes which drive the synthesis of mediating compounds necessary for the inflammatory process [38,39]. Perioperative administration of non-specific cyclooxygenase-inhibiting NSAIDs has been shown to consistently and significantly reduce immediate postoperative pain scores and opioid consumption [40]. Specifically, TJA patients who receive preoperative NSAIDs demonstrate reduced pain scores and reduced patient-controlled analgesia morphine consumption for the first 24 h post-surgery when compared to placebo [41].

NSAIDs, however, can induce potentially severe adverse gastrointestinal effects [42]. Specific COX-2-inhibiting drugs like celecoxib have been developed and are often preferred for their analgesic effects and reduced risk profile [14]. Numerous randomized-controlled trials (RCT) have confirmed the pain control efficacy of COX-2-specific inhibitors without showing any increase in surgical blood loss or other side effects compared to controls [43,44,45]. In the reviewed trials, administration both immediately before the first surgical incision as well as over the 24 h period before surgery exhibited significant positive outcomes postoperatively.

In addition to oral selective COX-2 inhibitors, topical forms of non-selective COX-inhibiting medications such as topical diclofenac and ibuprofen gel are frequently used [46]. Importantly, studies have observed that topical NSAIDs are less effective than systemic NSAIDs in terms of analgesia, but have a more favorable safety profile with the exception of local dermatologic side effects [47].

Acetaminophen is used synergistically with NSAIDs to combat surgical pain, and works by inhibiting the cyclooxygenase pathway. However, unlike NSAIDs, acetaminophen’s actions on the COX pathway are limited to the CNS, and the drug does not limit the portion of the inflammatory cascade that occurs peripherally [48]. It is widely used by physicians postoperatively as it is inexpensive, effective, well-researched, and has few side effects when compared to opioids [40].

Preoperative oral acetaminophen and intraoperative intravenous acetaminophen reduce pain scores and morphine consumption through the first three days after TJA [49,50,51]. Though there is some disagreement between studies regarding whether oral or intravenous acetaminophen is more effective, both forms improve pain outcomes and either can be used safely when clinically indicated [40,52].

### 3.3. NMDA Receptor Antagonists

Ketamine and dextromethorphan are non-competitive NMDA receptor antagonists. These medications inhibit the ability of CNS neurotransmitters to bind to NMDA receptors in afferent pain pathways, thus limiting CNS inflammatory and neuronal pain signaling [53]. These drugs also have known interactions with opioid, cholinergic, and monoamine receptors as well, which may contribute to observed effectiveness for surgical pain prevention [54,55].

Ketamine, originally developed as a general anesthetic, is well-researched and established for use in subanesthetic concentrations to treat chronic and acute pain, and has been shown to be exceptionally effective for opioid-tolerant patients [54,56,57]. Specifically, one meta-analysis of 20 RCTs showed that intravenous, low-dose ketamine administered preoperatively or intraoperatively reduced overall pain scores and opioid consumption in orthopedic surgery patients at 24 and 48 h postoperatively [58]. Similarly, another analysis examining the analgesic effectiveness of preoperative dextromethorphan demonstrated significant reductions in opioid consumption and pain scores over the first 24 h after surgery [59].

Despite extensive positive results regarding the effectiveness of NMDA receptor antagonists as surgical analgesics, there is still some controversy regarding their efficacy, as well as great variability between drug choice, dosage, and timing protocols between existing studies [60]. Despite this, the preponderance of the evidence points to NMDA receptor antagonists being exceptionally effective in procedures greater than 90 min in duration and those with more extensive tissue trauma, such as TJA [55,58]. Further study is warranted to define specific parameters regarding the role of drugs like ketamine in TJA and other major orthopedic procedures.

### 3.4. Intraarticular and Systemic Corticosteroids

Corticosteroids work as anti-inflammatory agents through the suppression of cellular immune function, thus reducing inflammatory pain and mitigating subsequent hyperalgesia [61,62]. A number of RCTs have exhibited evidence that both systemic and locally administered corticosteroids in the pre-, intra-, and postoperative periods effectively and safely reduce pain scores and opioid consumption after TJA [63,64]. One analysis of TJA outcomes showed that patients who received perioperative systemic corticosteroids (1–3 doses of dexamethasone, prednisone, hydrocortisone, or methylprednisone pre- and/or postoperatively) had lower active and resting pain scores, reduced opioid consumption, and no increase in infection or other complication risk compared to controls [64]. The addition of corticosteroids to periarticular local anesthetic injections improves the range of motion and functional status in early recovery stages, reduces pain scores, and reduces opioid consumption without increasing complication risk [65].

Perioperative corticosteroids in TJA have even been shown to exhibit some level of dose-responsiveness in their analgesic effectiveness. One retrospective cohort review of over 4200 TJA cases demonstrated that the patients who received two perioperative doses of 10 mg intravenous dexamethasone had improved outcomes compared to those who received one dose of the medication [66]. In that study, patients who received two doses exhibited reduced opioid consumption and self-assessed pain scores throughout the first three postoperative days compared to the single-dose group with comparable functional recoveries.

Corticosteroids do confer risk of adverse effects, including increased likelihood of infection due to immune suppression, delayed wound healing, and others [67]. However, these side effects are more commonly associated with prolonged systemic use, and short-course corticosteroids are a safe and effective way to reduce surgical pain and improve recovery metrics for most patients [64,65,67].

### 3.5. Gabapentin and Pregabalin

The gabapentinoids, gabapentin and pregabalin, are anticonvulsants used to treat neuropathic pain conditions such as neuropathy and fibromyalgia [68]. These medications bind to the alpha-2-delta subtype of presynaptic VGCCs, which reduces the concentration of calcium in presynaptic nerve terminals, thus decreasing the release of excitatory neurotransmitters, such as glutamate, in the CNS [69].

Gabapentinoids have become common off-label adjuncts to multimodal pain management for TJA patients, though evidence regarding their efficacy for reducing post-surgical pain and opioid consumption is mixed. Some analyses have indicated that gabapentin specifically has analgesic and opioid dependence-reducing qualities when applied both preoperatively and postoperatively [69,70,71,72,73]. Other studies, however, have suggested that the medication has no effect on postoperative pain or opioid consumption in TJA patients and that it may even lead to increased risk of adverse effects during recovery [74,75,76,77]. Due to the risk of side effects associated with gabapentin, especially when taken in combination with opioids, careful consideration should be applied when prescribing gabapentin off-label for surgical pain [75,78].

Pregabalin has moderate evidence supporting its efficacy, both with single and multidose perioperative administrations, for reducing post-TJA pain and opioid dependence [79]. Pregabalin is six times more potent than gabapentin and more rapidly effective, making it equally effective for neuropathic pain at much lower doses [27]. Pregabalin also exhibits several distinct advantages with regard to pharmacokinetic predictability and bioavailability when compared with gabapentin [80]. The American Academy of Orthopedic Surgeons (AAOS) recommends further study to determine pregabalin’s role in optimal pain management, but acknowledges the existing evidence supporting its positive effect on pain control [81].

## 4. Anesthetic Techniques and Postoperative Pain

Historically, general anesthesia has been the standard of care for patients undergoing major surgeries like TJA [82]. General anesthesia (GA) has several notable benefits and side effects, including anterograde and retrograde amnesia, intraoperative paralysis, absence of intraoperative patient anxiety, and greater control over the patient’s airway throughout the procedure [83,84]. However, other more targeted modalities have become increasingly common in recent decades, and research has indicated that various types of regional anesthesia are associated with improved pain-related and functional outcomes [82,84,85].

### 4.1. Neuraxial Anesthesia: Epidural and Spinal

Spinal and epidural anesthesia, collectively referred to as neuraxial anesthesia, involves delivering anesthetics and analgesics to the cerebrospinal fluid in the subarachnoid or epidural space of the lumbar spine [82]. Neuraxial anesthesia can be given for the duration of a procedure for durable pain control that persists into the postoperative period [84]. Epidural catheters may even remain in place after surgery for use as postoperative analgesia, with some evidence of improved outcomes compared to systemic opioid-based analgesia [86,87].

Several studies have demonstrated that spinal anesthesia is associated with less analgesic requirement in the post-anesthesia care unit (PACU) and shorter hospital stays compared to GA in several different types of orthopedic surgery. One study that examined outcomes for 17,690 patients who underwent primary TKA under either general or spinal anesthesia observed that the neuraxial anesthetic group had lower average postoperative pain scores, required less oral morphine equivalents, and had significantly fewer 30 and 90 day readmissions [88]. Numerous other studies have reported similar results, indicating that neuraxial anesthesia is associated with improved outcomes and a reduced risk of nearly all reported complications compared to GA [89,90,91]. This has led to a generally accepted consensus that neuraxial anesthesia should be preferred to GA when possible.

### 4.2. Peripheral Nerve Blockade

Peripheral nerve blocks (PNBs) are another highly effective adjunct in regional anesthesia commonly used in ambulatory procedures to localize anesthetic effects to a specific nerve or plexus. They can reduce postoperative pain and shorten inpatient hospitalizations when used in conjunction with GA compared to GA alone [92]. PNBs can be performed as single injections or through continuous delivery of anesthetic agent via a percutaneous catheter. The duration and density of the block can also be easily modulated based on the choice, concentration, and amount of local anesthetic agent used [93]. PNBs that can be used in TKA and THA include blocks of the obturator nerve, parasacral sciatic nerve, lumbar plexus-psoas, adductor canal, and other combination blocks such as the pericapsular nerve group (PENG) [94,95,96,97].

Based on moderate clinical evidence, PNBs appear to provide optimal outcomes compared to other anesthetic modalities in terms of reducing postoperative complications associated with TJA. In fact, PNB anesthesia is the current consensus recommendation for TJA from the International Consensus on Anesthesia-Related Outcomes after Surgery (ICAROS). This recommendation was based on a meta-analysis of 122 RCTs and observational studies involving over one million TJA patients [98]. That analysis found that PNB anesthesia was associated with reduced risk of serious postoperative complications such as cognitive dysfunction, cardiac complication, surgical site infection, respiratory failure, thromboembolism, and blood transfusions. In a separate study that included over one million TJA patients, the use of PNBs resulted in a 16.2% and 12.7% reduction in postoperative opioid consumption for THA and TKA patients, respectively [99].

Although this form of anesthesia has demonstrated exceptional efficacy and safety, when used for continuous postoperative analgesia PNB may delay patients’ progression to physical therapy and impact their overall recovery due to lower extremity motor control interference. Therefore, the benefits and effectiveness of continuous PNBs should be weighed against the potential consequences of delayed mobilization. Nonetheless, PNBs have few contraindications and are widely regarded as the preferred modality of anesthesia for TJA procedures.

### 4.3. Local Infiltration Periarticular Injection

Local infiltration anesthesia (LIA) involves the intraoperative direct application of at least one anesthetic agent to the periosteum and joint capsule to reduce postoperative pain, mitigate opioid consumption, and promote early mobilization. LIA medication mixtures commonly include combinations of local anesthetic, epinephrine, NSAIDs, opioids, and steroids, and can be used in combination with GA, neuraxial anesthesia, or PNB.

Several studies have observed that perioperative LIA is effective for reducing short-term postoperative pain, length of hospital stays, and opioid consumption associated with TJA [100]. Continued postoperative LIA has also demonstrated equivalent pain control as well as reduced nausea and improved range of motion at 24 and 48 h after surgery when compared to postoperative epidural analgesia [101]. Therefore, continuous postoperative LIA may be a preferable option for postoperative pain control because it interferes less with lower extremity motor control which likely leads to expedited mobilization.

Though LIA techniques are commonly implemented today and most of the evidence supports their efficacy, there is still a lack of consensus on the optimal protocol [102]. Because there is no clinical consensus on the best mixtures and methods for LIA application, the existing studies lack uniformity regarding medications used and timing of application, leading to some inherent uncertainty in conclusions. Further research on optimal protocols is necessary to establish standardized guidelines for clinical application of LIA.

## 5. Non-Pharmacologic and Novel Strategies for Surgical Pain Management

### 5.1. Perioperative Physical Therapy and Exercise

Given the well-characterized relationship between physical activity and cardiovascular health on generalized health outcomes, numerous studies have examined the efficacy of short-term physical preparation programs for improving surgical outcomes. Several of these investigations have shown that preoperative physical therapy can have positive effects on post-surgical pain, length of hospital stay, functional recovery, and opioid consumption; however, reported results have been inconsistent [103,104,105,106]. Contradictory conclusions between studies could be due to variable program intensity, patient compliance, and program duration. Given the variability in reported efficacy of these programs, their cost may constitute a potential limitation to their broader implementation. More research is needed to determine the analgesic efficacy of preoperative rehabilitation programs and the overall cost-effectiveness of implementation.

Postoperatively, physical therapy is a well-established and proven component of the surgical recovery process, essential for reducing chronic pain and increasing functionality [107]. Earlier postoperative physical therapy after TJA is associated with shortened hospital stays, reduced care costs, improved functional outcomes, as well as reductions in opioid consumption and pain scores [108,109,110]. In fact, patients who start physical therapy within 24 h of their TJA procedure experience lower rates of 30-day readmission and sooner achievement of functional autonomy compared to those who do not [111,112]. Therefore, early postoperative mobilization and physical therapy following TJA is one of the most effective and highly recommended solutions to chronic surgical pain.

### 5.2. Cryotherapy

Cryotherapy appears to exhibit some benefit in terms of postoperative pain and recovery of function; however, its efficacy is still disputed due to a paucity of reliable research [113]. Cryotherapeutic analgesia facilitates a drop in the intraarticular temperature [114]. The drop in intraarticular and surrounding tissue temperature theoretically leads to reduced local nerve conduction, inflammation, tissue edema, blood flow, and ultimately less postoperative pain, reduced swelling, and improved mobility [113].

While the evidence regarding its efficacy is not definitive, aggregated data on the use of cryotherapy for surgical recovery has suggested that it may provide marginal benefits in terms of pain reduction, decreased blood loss, and mobility recovery [115]. Though the overall quality of the evidence is considered low, partially due to the lack of standardized implementation protocols, postoperative cryotherapy is commonly implemented and in accordance with AAOS clinical practice guidelines for musculoskeletal extremity surgeries [81]. This is due to the relatively few potential side effects of local cryotherapy. However, more research on this analgesic option is necessary to determine its optimal role in recovery.

### 5.3. Percutaneous Peripheral Nerve Stimulation

Percutaneous peripheral nerve stimulation (PNS) is a non-pharmacologic analgesic therapy that involves the use of ultrasound to place perineural electrodes for the purpose of electrically stimulating a particular nerve. The most commonly accepted theory describing the analgesic mechanism behind percutaneous PNS is known as gate control theory, which postulates that when peripheral afferent nerve fibers are activated by non-noxious stimuli, it impedes the simultaneous transfer of pain signaling [116]. Neural stimulation for analgesia has been in use for decades in patients with chronic pain and has some clinical research to support its efficacy; however, many of the studies examining its effectiveness have very small study cohorts, bringing the generalizability of their observations into question [116,117,118,119].

The most significant limitation in its broader testing and application, particularly with surgical patients, is how invasive the technique is. PNS involves the surgical placement of percutaneous hardware in the vicinity of nerves, presenting significant potential for consequences such as infection and nerve damage. In order to mitigate these types of risks, a potential alternative could involve the use of transcutaneous electrical nerve stimulation (TENS) via skin electrodes. However, the efficacy of this alternative has even less evidentiary support and may not achieve even a marginal positive effect [116].

The cost of PNS and TENS protocols may constitute an additional limiting factor to their application given the limited evidence regarding their efficacy. Electrical stimulation techniques could present a viable option for post-TJA pain control in the future. However, extensive further research is required prior to their widespread implementation in a pain management protocol.

## 6. Evidence-Based Opioid Sparing Protocol Example

**Figure 1:**
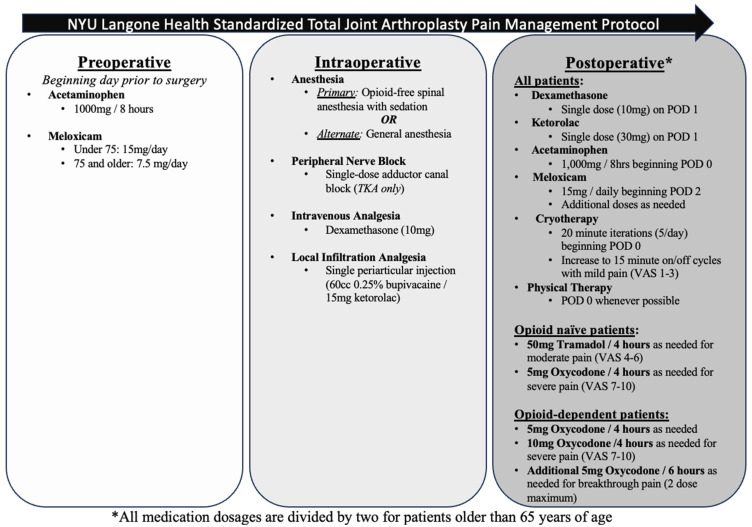
NYU Langone Health multimodal pain management protocol (MPMP).

Our institution uses a standardized multimodal pain management protocol (MPMP) for all patients who undergo TJA (Figure 1). This protocol is modified based on a number of patient-specific factors including age, medical comorbidities, and history of opioid-dependence. Preoperatively, patients are prescribed acetaminophen (1000 mg every 8 h) and meloxicam (single 15 mg dose) starting one day prior to surgery.

During the procedure, an opioid-free spinal anesthetic is administered (either ropivacaine or mepivacaine) with sedation, except in cases in which the patient refuses spinal anesthesia, spinal anesthesia is unsuccessful, or contraindicated. In these cases, general anesthesia is used. Intraoperative medications include an intravenous dose of dexamethasone (10 mg) for analgesia, in addition to a periarticular injection comprised of 60 cc 0.25% bupivacaine and 15 mg ketorolac, infiltrated into the capsule and periosteum. Furthermore, all primary elective TKA patients are given a single-dose adductor canal block.

Beginning on postoperative day (POD) 0, cold therapy is administered five times per day for 20 min at a time. Patients also begin physical therapy on the same day as their operation if they are awake prior to 1800 that day. In cases where patients do not wake up until after 1800 on the evening of their surgery, they begin physical therapy the following morning.

Patients younger than 65 years receive 1000 mg oral acetaminophen every 8 h starting on postoperative day 0. Patients also receive a single 10 mg dose of dexamethasone and one 30 mg dose of ketorolac on POD 1. Daily 15 mg meloxicam is initiated on POD 2. If patients require further pain control, additional doses of medication are provided. For patients experiencing mild pain (visual analog scale (VAS) score of 1 to 3), cold therapy is increased to 15 min on and 15 min off with skin evaluations. For moderate pain (VAS 4 to 6), non-opioid-dependent patients are prescribed either 50 mg Tramadol every four hours as needed, and for severe pain (VAS 7 to 10) 5 mg Oxycodone is given every four hours as needed. Opioid-dependent patients receive 5 mg oxycodone every four hours as needed for mild to moderate pain; this dose is increased to 10 mg for severe pain. Another 5 mg Oxycodone dose can be given every 6 h for breakthrough pain for a maximum of two doses. For patients over 65 years, all dosages are divided in half.

For chronic opioid users, patients with allergies or medical conditions interfering with the standardized pain protocol, or those with pain not well-controlled under the protocol, a 24 h pain management consult service is available.

## 7. Conclusions

Postoperative pain is a significant challenge for many hip and knee arthroplasty patients that inhibits recovery, reduces patient satisfaction, and increases healthcare costs. Multimodal pain management strategies are now the standard of care for mitigating patient suffering and secondary complications following TJA procedures. Despite significant emphasis on pain control following TJA, a high degree of variability remains among pain-response strategies. These different approaches to perioperative pain management are likely secondary to institutional limitations and differences in patient populations. More targeted research is necessary to better identify which patients are at risk of experiencing maladaptive pain after TJA and to elucidate more standardized treatment strategies.

## Data Availability

No data were utilized in the creation of this manuscript.
